# Seasonal dynamics and burden of respiratory syncytial virus, influenza, and COVID-19 in Brazil: a national surveillance study (2019–2023)

**DOI:** 10.7189/jogh.16.04251

**Published:** 2026-08-28

**Authors:** Guilherme Silva Julian, Julia Regazzini Spinardi, Ana Paula Flora, Srinivas Rao Valluri, Florence Lefebvre d'Hellencourt, Caihua Liang, Elizabeth Begier, Thaís Zamboni Berra, Lucas Vieira Cortez, Renato Vitorasso, Moe Hein Kyaw

**Affiliations:** 1 Pfizer, São Paulo, São Paulo, Brazil; 2 Pfizer, New York, USA; 3 IQVIA, São Paulo, São Paulo, Brazil

**Keywords:** respiratory syncytial virus, influenza virus, COVID-19, seasonality, surveillance data analysis, Brazil

## Abstract

**Background:**

The burden and seasonal patterns of respiratory syncytial virus (RSV), influenza, and COVID-19 remain poorly characterised in Brazil and other Latin American countries. Understanding their epidemiological patterns is essential to inform public health interventions and preventive strategies. We aimed to assess respiratory virus seasonality using national surveillance data to guide vaccination timing and strengthen preparedness strategies in Brazil.

**Methods:**

We conducted a retrospective, observational, descriptive population-based study using national surveillance data collected through the SIVEP system between 2019 and 2023. We analysed confirmed cases, hospitalisations, and deaths attributed to RSV, influenza, and COVID-19. We performed time-series decomposition and seasonal trend analysis using the multiple seasonal-trend decomposition *via* locally estimated scatterplot smoothing method. We calculated monthly mean values for each aetiology component to assess their seasonal burden.

**Results:**

There were 69,389 RSV, 45,881 influenza, and 2,299,712 COVID-19 cases among the laboratory-confirmed severe acute respiratory infection (SARI) cases reported in SIVEP. Each year, more than 90% of RSV diagnoses were reported in children aged 0–10 years, while influenza and COVID-19 were more common among older adults. The proportion of deaths in children related to RSV increased from 19.2% in 2020 to 62.0% in 2023. Seasonal peaks occurred from March to June for RSV, influenza cases peaked in January, while COVID-19 showed less consistent seasonality. Geographic variation was observed, with higher case concentrations in the Southeast and South regions of Brazil.

**Conclusions:**

The burden of respiratory viruses among SARI cases reported in the national surveillance system in Brazil differed by pathogen, age group, region, and season, and may be influenced by variations in testing strategies. Strengthening diagnostic capacity and surveillance is critical, particularly for adult RSV cases, which are likely underreported due to limited testing and reduced diagnostic sensitivity. Seasonal vaccination programmes, such as those for influenza, remain essential and may be further enhanced by integrating new preventive measures, such as RSV vaccination for older adults and maternal immunisation to protect infants.

Respiratory virus infections are a major cause of morbidity and mortality in Brazil and globally [1]. Respiratory syncytial virus (RSV), influenza, and COVID-19 are among the most important aetiological agents of viral respiratory diseases due to their persistent circulation, high transmissibility, and significant impact on public health [1]. These three pathogens are responsible for substantial hospitalisations and deaths across all age groups. While anyone can be infected, at-risk groups such as children, older adults, and individuals with underlying medical conditions account for the majority of the disease burden [2]. They are also more vulnerable to severe complications, which can lead to hospitalisation and, in many cases, death [3].

Recent years have seen the emergence of the so-called ‘tripledemic’, characterised by the concurrent circulation of RSV, influenza, and COVID-19 [4]. This phenomenon has placed substantial strain on health systems, particularly in regions with limited surveillance infrastructure and variable access to diagnostic testing [5]. Brazil’s mandatory national epidemiological surveillance information system (*Sistema de Informação da Vigilância Epidemiológica da Gripe *(SIVEP-Gripe)) provides an important source of data for assessing the epidemiology of severe acute respiratory infections (SARI) attributed to these viruses, offering insights that are also relevant for other countries in the Americas [6].

Despite advances in vaccination and preventive measures, critical gaps remain in the characterisation of disease burden and seasonality for RSV, influenza, and COVID-19 in Latin America [7], hindering the development of targeted public health interventions and the optimisation of vaccination strategies. Brazil’s recent inclusion of maternal RSV vaccination in its national immunisation programme exemplifies how approaches to prevention evolve over time, but also points to a need for robust epidemiological evidence to guide the implementation of such policy [4].

We aimed to describe the clinical and demographic profile, mortality, and hospitalisation burden of SARI cases attributed to RSV, influenza, and COVID-19 in Brazil from 2019 to 2023. With this in mind, we formulated the following research questions:

1. How did the epidemiological burden of RSV, influenza, and COVID‑19 compare across age groups with respect to cases, hospitalisations, and deaths?
2. How did the distribution and seasonality of these viruses differ across Brazil’s five macroregions?
3. How did the temporal patterns of virus circulation change from 2019 to 2023, encompassing the pre‑pandemic, pandemic, and post‑emergency phases of COVID‑19?

By analysing national surveillance data and seasonal trends, we wanted to inform the timing of vaccination campaigns, resource allocation, and preparedness strategies for respiratory virus outbreaks in Brazil and other countries in the Americas.

## METHODS

We conducted a retrospective, observational, descriptive study using national surveillance data on SARI cases attributed to RSV, influenza, and COVID-19 reported in SIVEP-Gripe between January 2019 and December 2023.

### Population

We included all laboratory-confirmed cases of SARI due to RSV, influenza, or SARS-CoV-2 that were reported in SIVEP-Gripe between January 2019 and December 2023, regardless of age or diagnostic method (*e.g.* reverse transcription polymerase chain reaction, rapid antigen testing, and viral panel testing). We applied no additional exclusion criteria.

The analysis was based on cases reported to SIVEP based on routine standard-of-care testing [8]. The clinical case definition used for reporting to SIVEP follows Brazil’s national criteria for SARI, which align with World Health Organization standards [9]. Specifically, a case of SARI is defined as an individual who presents flu-like symptoms such as fever, cough, sore throat, or other respiratory symptoms, together with at least one sign of severity (*e.g.* dyspnoea, persistent chest pain, oxygen saturation below 95% on room air, or cyanosis of the lips or face).

### Data source

We obtained data from the publicly accessible SIVEP-Gripe database, which is managed by the Brazilian Ministry of Health [10]. The database contains anonymised records of all reported SARI cases, is updated regularly, and is available through the OpenDataSUS platform [11]. Data are managed in compliance with Brazil’s General Data Protection Law (law no. 13,709/2018).

SIVEP‑Gripe primarily captures hospitalised SARI cases and SARI-related deaths. However, not all records represent hospital admissions, as deaths may occur outside hospital settings and cases may be reported prior to or without admission. We, therefore, determined hospitalisation status using the corresponding SIVEP-Gripe variable, rather than assuming that all reported cases involved hospital admission.

### Testing variability and selection bias

SIVEP-Gripe does not provide standardised indicators of testing intensity. We restricted the analyses to laboratory-confirmed cases and interpreted the findings considering differences in diagnostic coverage but did not adjust for testing intensity. For RSV, we reported results using absolute counts, proportions, and seasonal timing, rather than population rates.

### Outcomes

Primary outcomes included the frequency and demographic distributions of laboratory-confirmed RSV, influenza, and COVID-19 cases, hospitalisations, and deaths. Secondary outcomes included case fatality rates (CFRs), calculated as the proportion of deaths among laboratory-confirmed cases, and hospitalisation rates per 100,000 population, stratified by age group, year, and region. For RSV, we did not calculate hospitalisation rates, mortality rates or CFRs due to limited testing and diagnostic coverage among adults.

We identified hospitalisations using the hospitalisation status recorded in the SIVEP database under the variable ‘hospitalised’. Because not all reported SARI cases were hospitalised, we analysed hospitalisation as a separate outcome rather than an inherent characteristic of the dataset. We identified deaths through the outcome variable *‘*evolution’, which includes deaths occurring both in hospital and outside hospital settings. These records represent deaths among confirmed SARI cases, but do not establish that the pathogen was the primary or direct cause of death. SIVEP does not adjudicate causality, and deaths may involve patients with multiple comorbidities or coinfections.

### Statistical analysis

We summarised demographic characteristics by age (primarily stratified into ten-year intervals), sex, race or ethnicity, and region. We also conducted prespecified analyses for all ages combined, children younger than two years, adults aged 60 years or older, and adults aged 70 years or older. We assessed geographic variation using ArcGIS, version 10.5 (Esri, Redlands, California, USA) and official Brazilian territorial shapefiles [12]. We calculated absolute and relative frequencies of cases, hospitalisations, and deaths.

For influenza and COVID-19, we calculated CFRs and hospitalisation rates per 100,000 population, with 95% confidence intervals (CIs). We did not calculate CFRs, mortality rates, or hospitalisation rates for RSV due to substantial under-ascertainment in adults and highly selective testing among children, which would make the metric epidemiologically misleading. To evaluate the contribution of each virus to hospitalisations and deaths, we calculated the proportion of events attributable to RSV, influenza, or COVID-19 within each age group. We divided the number of events attributed to each virus by the total number of events attributed to all three viruses and multiplied the result by 100. We restricted the analyses to laboratory-confirmed diagnoses and did not attribute events among untested cases to pathogens.

### Seasonality analysis

We assessed seasonality using monthly raw counts of laboratory-confirmed cases and hospitalisations. We did not use rates or proportions because SIVEP-Gripe lacks reliable denominators for testing intensity or total number of tests by pathogen, region, or age group. Therefore, the decomposition reflects seasonal patterns in reported surveillance activity, not incidence.

We performed decomposition using the multiple seasonal-trend decomposition *via* locally estimated scatterplot smoothing (MSTL) method with a 12‑month period. We selected this approach to accommodate pathogen-specific and non-stationary seasonal patterns, which changed substantially during the COVID-19 pandemic, making traditional single-season decomposition methods less suitable for capturing the complex temporal variations in respiratory virus circulation observed during the study period [13-15].

We applied the robust option of the MSTL decomposition, which uses locally estimated scatterplot smoothing to reduce the influence of extreme COVID-19 peaks. Decomposition diagnostics included visual evaluation of the seasonal, trend, and residual components to verify stability and determine whether the 2020–2021 structural break introduced artificial seasonality. We used MSTL results solely for descriptive interpretation.

For visualisation, we extracted the annual seasonal component from the MSTL models and averaged the monthly seasonal values across the study years. We displayed these averages using heatmaps to identify months contributing most to annual increases in hospitalisations due to respiratory viruses and to compare seasonal patterns across Brazil’s five regions: North, Northeast, South, Southeast, and Midwest [16,17].

## RESULTS

The SIVEP-Gripe surveillance system recorded 69,389 RSV cases, 45,881 influenza cases, and 2,299,712 COVID-19 cases in Brazil between 2019 and 2023. RSV diagnoses were predominantly reported in children aged 0–10 years (92.9%), with a median age of 0 years (interquartile range (IQR) = 0–1). Influenza and COVID-19 diagnoses were more frequently reported among older adults, with median ages of 40 years (IQR = 7–70) and 59 years (IQR = 45–73), respectively. The Southeast and South regions accounted for the highest proportion of cases for all three viruses (**Table 1**).

**Table 1:** Demographic and geographic characteristics of cases of RSV, influenza and COVID-19 in Brazil, 2019–2023*

	RSV	Influenza	COVID-19
**Age**			
Valid observations	69,389 (100.0)	45,881 (100.0)	2,299,712 (100.0)
x̄ (SD)	4.7 (16.2)	40.6 (31.7)	58.0 (19.6)
Mdn (range; IQR)	0.0 (0.0–109.0; 0.0–1.0)	40.0 (0.0–110.0; 7.0–70.0)	59.0 (0.0–119.0; 45.0–73.0)
**Gender**			
Valid observations	69,378 (99.9)	45,874 (99.9)	2,299,432 (99.9)
Female	31,537 (45.5)	23,972 (52.3)	1,039,005 (45.2)
Male	37,841 (54.5)	21,902 (47.7)	1,260,427 (54.8)
**Race or colour**			
Valid observations	56,494 (81.4)	37,607 (81.9)	1,880,327 (81.7)
White	29,646 (52.5)	20,475 (54.4)	983,958 (52.3)
Black	1,796 (3.2)	1,645 (4.4)	97,284 (5.2)
Brown	24,387 (43.2)	14,961 (39.8)	771,904 (41.1)
Asian	277 (0.5)	361 (1.0)	22,596 (1.2)
Indigenous	388 (0.7)	165 (0.4)	4,585 (0.2)
**Region of residence**			
Valid observations	69,389 (100.0)	45,881 (100.0)	2,299,712 (100.0)
North	3,370 (4.9)	1,916 (4.2)	152,648 (6.6)
Northeast	9,540 (13.7)	8,709 (19.0)	381,812 (16.6)
South	19,034 (27.4)	8,825 (19.2)	392,460 (17.1)
Southeast	28,930 (41.7)	20,988 (45.7)	1,140,914 (49.6)
Midwest	8,515 (12.3)	5,443 (11.9)	231,878 (10.1)

IQR – interquartile range, Mdn – median, RSV – respiratory syncytial virus, SD – standard deviation, x̄ – mean

*Presented as n (%) unless specified otherwise.

### Temporal trends and age distribution

The number of RSV cases increased more than fourfold between 2019 and 2023, from 6,225 cases to 28,246. The proportion of RSV cases among children aged 0–10 years also increased, from 90.1% in 2019 to 95.7% in 2023, while diagnoses in older age groups remained consistently low (**Table 2**). Influenza cases fluctuated annually, with increases in 2021 and 2022, with children aged 0–10 years and adults aged 61 years or older accounting for the largest proportions of reported cases. COVID-19 cases peaked in 2020 and 2021, during the initial years of the pandemic, with adults aged 61–80 years accounting for the largest proportion of reported cases (**Table 2**).

**Table 2:** Frequency and age distribution of RSV, influenza, and COVID-19 cases in Brazil, by year of notification, 2019–2023*

	2019	2020	2021	2022	2023	Overall
**RSV**						
Frequency	6,225 (9.0)	1,725 (2.5)	14,264 (20.5)	18,929 (27.3)	28,246 (40.7)	69,389 (100.0)
Age group in years						
*0–10*	5,611 (90.1)	1,315 (76.2)	13,050 (91.5)	17,462 (92.2)	27,040 (95.7)	64,478 (92.9)
*11–20*	65 (1.0)	17 (1.0)	141 (1.0)	110 (0.6)	188 (0.7)	521 (0.8)
*21–35*	100 (1.6)	60 (3.5)	108 (0.8)	110 (0.6)	82 (0.3)	460 (0.7)
*36–45*	56 (0.9)	55 (3.2)	96 (0.7)	80 (0.4)	76 (0.3)	363 (0.5)
*46–60*	117 (1.9)	78 (4.5)	210 (1.5)	204 (1.1)	162 (0.6)	771 (1.1)
*61–80*	163 (2.6)	143 (8.3)	421 (3.0)	590 (3.1)	426 (1.5)	1,743 (2.5)
*≥81*	113 (1.8)	57 (3.3)	238 (1.7)	373 (2.0)	272 (1.0)	1,053 (1.5)
**Influenza**						
Frequency	6,847 (14.9)	2,366 (5.2)	11,803 (25.7)	12,014 (26.2)	12,851 (28.0)	45,881 (100.0)
Age group in years						
*0–10*	2,088 (30.5)	668 (28.2)	1,815 (15.4)	3,223 (26.8)	5,509 (42.9)	13,303 (29.0)
*11–20*	385 (5.6)	154 (6.5)	588 (5.0)	849 (7.1)	1,268 (9.9)	3,244 (7.1)
*21–35*	859 (12.5)	416 (17.6)	1,416 (12.0)	953 (7.9)	942 (7.3)	4,586 (10.0)
*36–45*	690 (10.1)	271 (11.5)	835 (7.1)	615 (5.1)	893 (6.9)	3,304 (7.2)
*46–60*	1,123 (16.4)	313 (13.2)	1,174 (9.9)	1,072 (8.9)	1,174 (9.1)	4,856 (10.6)
*61–80*	1,224 (17.9)	382 (16.1)	3,408 (28.9)	3,188 (26.5)	1,995 (15.5)	10,197 (22.2)
*≥81*	478 (7.0)	162 (6.8)	2,567 (21.7)	2,114 (17.6)	1,070 (8.3)	6,391 (13.9)
**COVID-19**						
Frequency	NA	743,239 (32.3)	1,254,807 (54.6)	247,299 (10.7)	54,366 (2.4)	2,299,712 (100.0)
Age group in years						
*0–10*	NA	11,013 (1.5)	14,790 (1.2)	18,636 (7.5)	8,323 (15.3)	52,762 (2.3)
*11–20*	NA	6,861 (0.9)	9,231 (0.7)	4,560 (1.8)	1,137 (2.1)	21,789 (0.9)
*21–35*	NA	56,677 (7.6)	111,129 (8.9)	15,385 (6.2)	2,995 (5.5)	186,187 (8.1)
*36–45*	NA	88,711 (11.9)	193,133 (15.4)	14,383 (5.8)	2,773 (5.1)	299,000 (13.0)
*46–60*	NA	184,837 (24.9)	380,261 (30.3)	32,326 (13.1)	6,125 (11.3)	603,549 (26.2)
*61–80*	NA	285,707 (38.4)	418,475 (33.3)	88,967 (36.0)	17,879 (32.9)	811,028 (35.3)
*≥81*	NA	109,433 (14.7)	127,788 (10.2)	73,042 (29.5)	15,134 (27.8)	325,397 (14.1)

NA – not applicable, RSV – respiratory syncytial virus

*Presented as n (%) unless specified otherwise.

**Figure 1:**
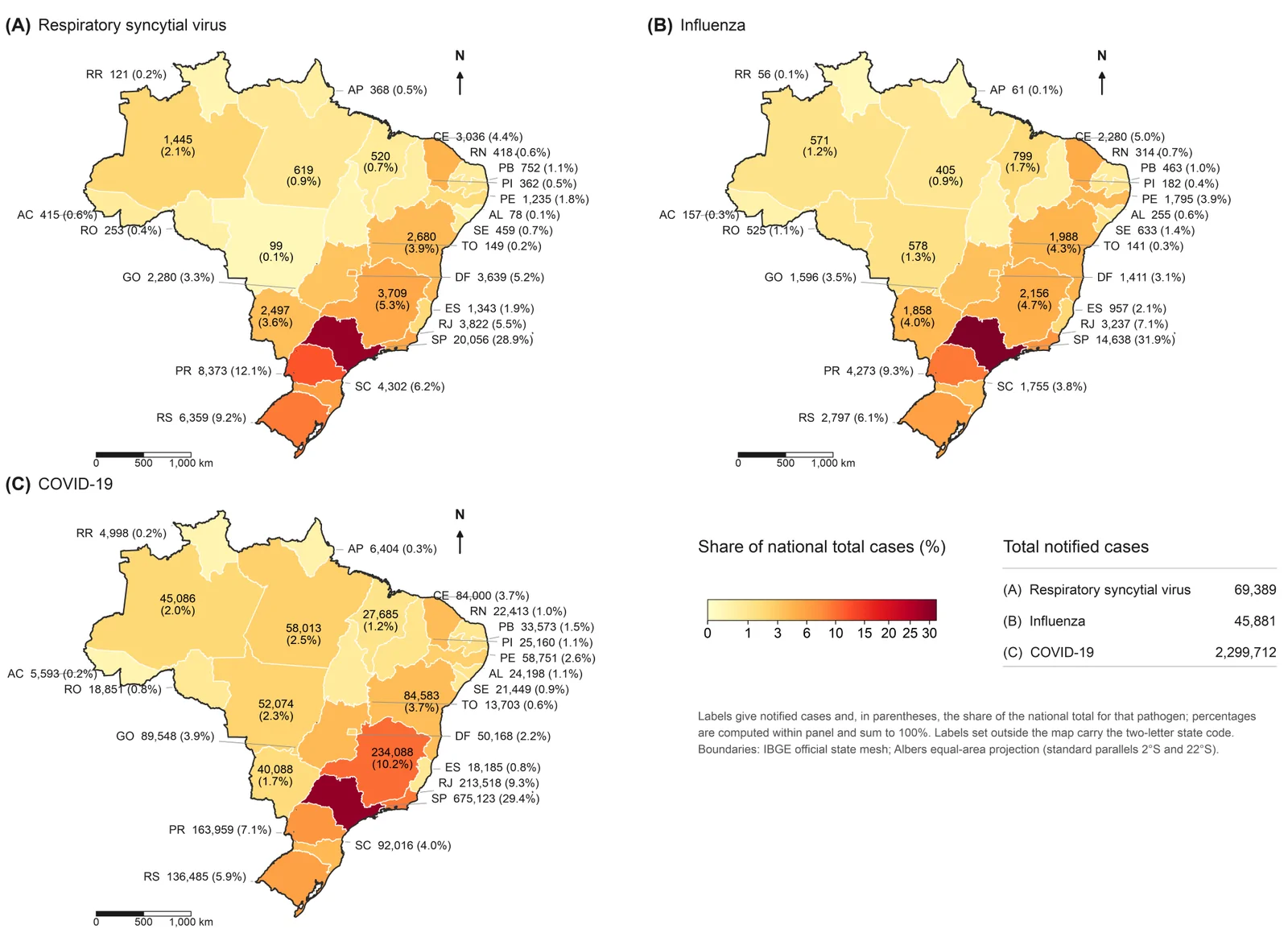
Geographic variation in the frequency of respiratory syncytial virus, influenza, and COVID-19 according to federative unit of residence in Brazil, 2019–2023. **Panel A.** Respiratory syncytial virus cases. **Panel B**. Influenza cases. **Panel C.** COVID-19 cases.

States in the Southeast and South regions accounted for the largest proportion of reported cases, while states in the North region generally accounted for smaller proportions (**Figure 1**). For RSV, São Paulo in the Southeast region (n = 20,056, 28.9%) and Paraná in the South region (8,373, 12.1%) recorded the largest numbers of cases; Mato Grosso in the Midwest region (n = 99, 0.1%) and Alagoas in the Northeast region (n = 418, 0.6%) recorded the smallest numbers of notified RSV cases.

### Hospitalisation and mortality

**Figure 2:**
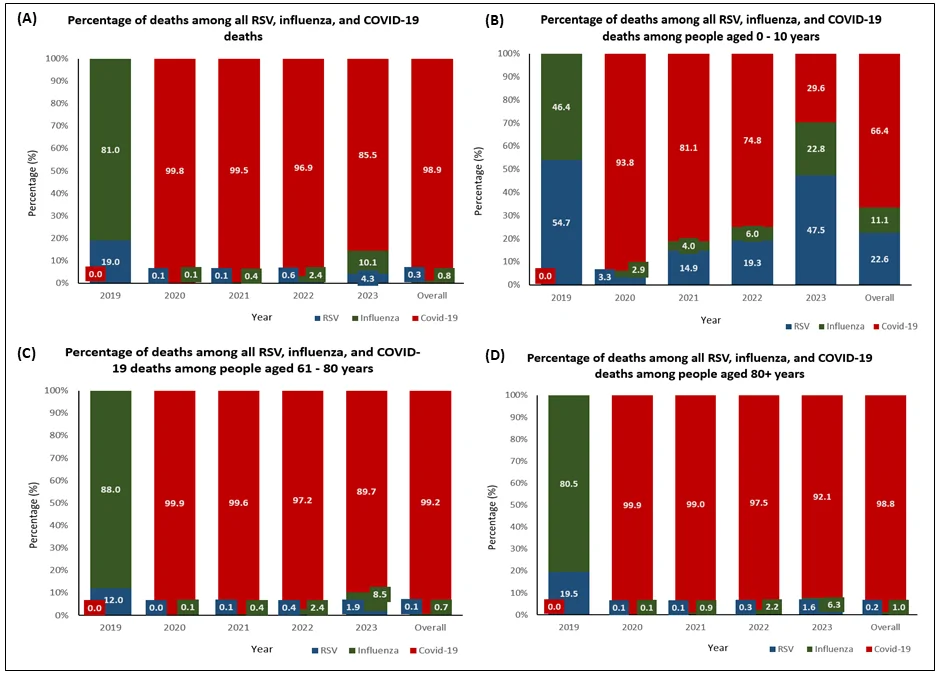
Percentage distribution of deaths attributed to respiratory syncytial virus, influenza, and COVID-19 across age groups in Brazil, 2019–2023. **Panel A.** Distribution of deaths across all age groups. **Panel B**. Distribution of deaths among children aged 0–10 years. **Panel C.** Distribution of deaths among adults aged 61–80 years. **Panel D.** Distribution of deaths among adults aged 81 years or older*.*

Hospitalisations and deaths attributed to RSV, influenza, and COVID-19 showed distinct age-related patterns. RSV accounted for most hospitalisations among children aged 0–10 years, while the proportion of RSV-attributed deaths increased from 52.2% in 2019 to 62.0% in 2023. Influenza deaths were more evenly distributed between children and older adults. COVID-19 mortality was concentrated among individuals aged 61 years or older, with individuals aged 81 years or older accounting for more than 50% of reported deaths (**Figure 2**, **Table 3**). Detailed age-specific percentages of deaths per year are provided in Table S2 in the **Online Supplementary Document**.

**Table 3:** Deaths and mortality rates among cases of respiratory syncytial virus, influenza, and COVID-19 in Brazil, by age group and year of notification, 2019–2023*

	2019	2020	2021	2022	2023	Overall
**RSV†**						
Overall	270 (100.0)	125 (100.0)	359 (100.0)	423 (100.0)	484 (100.0)	1,661 (100.0)
Deaths stratified by age groups						
*0–10*	141 (52.2)	24 (19.2)	136 (37.9)	165 (39.0)	300 (62.0)	766 (46.1)
*11–20*	2 (0.7)	0 (0.0)	8 (2.2)	3 (0.7)	6 (1.2)	19 (1.1)
*21–35*	16 (5.9)	6 (4.8)	9 (2.5)	7 (1.7)	7 (1.4)	45 (2.7)
*36–45*	9 (3.3)	8 (6.4)	12 (3.3)	13 (3.1)	6 (1.2)	48 (2.9)
*46–60*	26 (9.6)	16 (12.8)	35 (9.7)	35 (8.3)	17 (3.5)	129 (7.8)
*61–80*	45 (16.7)	40 (32.0)	91 (25.3)	109 (25.8)	82 (16.9)	367 (22.1)
*≥81*	31 (11.5)	31 (24.8)	68 (18.9)	91 (21.5)	66 (13.6)	287 (17.3)
**Influenza**						
Overall	1,152 (100.0)	311 (100.0)	1,711 (100.0)	1,596 (100.0)	1,138 (100.0)	5,908 (100.0)
Deaths stratified by age group						
*0–10*	122 (10.6)	21 (6.8)	37 (2.2)	51 (3.2)	144 (12.7)	375 (6.3)
*11–20*	31 (2.7)	11 (3.5)	26 (1.5)	23 (1.4)	55 (4.8)	146 (2.5)
*21–35*	78 (6.8)	28 (9.0)	68 (4.0)	56 (3.5)	52 (4.6)	282 (4.8)
*36–45*	124 (10.8)	24 (7.7)	80 (4.7)	55 (3.4)	82 (7.2)	365 (6.2)
*46–60*	338 (29.3)	60 (19.3)	184 (10.8)	185 (11.6)	168 (14.8)	935 (15.8)
*61–80*	331 (28.7)	105 (33.8)	668 (39.0)	632 (39.6)	370 (32.5)	2,106 (35.6)
*≥81*	128 (11.1)	62 (19.9)	648 (37.9)	594 (37.2)	267 (23.5)	1,699 (28.7)
Mortality rate per 100,000 population (95% CI) stratified by age groups						
*0–10*	0.4 (0.3–0.5)	0.1 (0.0–0.1)	0.1 (0.1– 0.2)	0.2 (0.1–0.2)	0.5 (0.4–0.6)	1.3 (1.1–1.4)
*11–20*	0.1 (0.1–0.1)	0.0 (0.0–0.0)	0.1 (0.1–0.1)	0.1 (0.0–0.1)	0.2 (0.1–0.2)	0.5 (0.4–0.6)
*21–35*	0.1 (0.1–0.2)	0.1 (0.0–0.1)	0.1 (0.1–0.2)	0.1 (0.1–0.1)	0.1 (0.1–0.1)	0.6 (0.5–0.7)
*36–45*	0.3 (0.3–0.4)	0.1 (0.0–0.1)	0.2 (0.2–0.3)	0.2 (0.1–0.2)	0.3 (0.2–0.3)	1.1 (1.0–1.3)
*46–60*	0.9 (0.8–1.0)	0.2 (0.1–0.2)	0.5 (0.4– 0.6)	0.5 (0.4–0.6)	0.4 (0.4–0.5)	2.5 (2.4–2.7)
*61–80*	1.3 (1.1–1.4)	0.4 (0.3–0.5)	2.6 (2.4–2.8)	2.4 (2.2–2.6)	1.4 (1.3–1.6)	8.1 (7.8–8.5)
*≥81*	3.2 (2.7–3.8)	1.6 (1.2–1.9)	16.3 (15.0–17.6)	14.9 (13.7–16.1)	6.7 (5.9–7.5)	42.7 (40.7–44.8)
**COVID-19**						
Overall deaths	-	232,834 (100.0)	384,076 (100.0)	63,649 (100.0)	9,651 (100.0)	690,210 (100.0)
Deaths stratified by age groups						
*0–10*	NA	679 (0.3)	743 (0.2)	641 (1.0)	187 (1.9)	2,250 (0.3)
*11–20*	NA	574 (0.2)	761 (0.2)	265 (0.4)	55 (0.6)	1,655 (0.2)
*21–35*	NA	4,942 (2.1)	13,476 (3.5)	1,287 (2.0)	214 (2.2)	19,919 (2.9)
*36–45*	NA	10,856 (4.7)	31,474 (8.2)	1,923 (3.0)	360 (3.7)	44,613 (6.5)
*46–60*	NA	38,208 (16.4)	94,955 (24.7)	6,700 (10.5)	1,023 (10.6)	140,886 (20.4)
*61–80*	NA	114,466 (49.2)	172,557 (44.9)	25,851 (40.6)	3,926 (40.7)	316,800 (45.9)
*≥81*	NA	63,109 (27.1)	70,110 (18.3)	26,982 (42.4)	3,886 (40.3)	164,087 (23.8)
Mortality rate per 100,000 population (95% CI) stratified by age groups						
*0–10*	NA	2.3 (2.1–2.5)	2.5 (2.4–2.7)	2.2 (2.0–2.4)	0.6 (0.5–0.7)	7.7 (7.4–8.0)
*11–20*	NA	2.0 (1.7–2.2)	2.7 (2.5–2.9)	0.9 (0.8–1.0)	0.2 (0.1–0.2)	5.8 (5.6–6.1)
*21–35*	NA	10.6 (10.3–10.9)	28.8 (28.3–29.3)	2.7 (2.6–2.9)	0.5 (0.4–0.5)	42.6 (42.0–43.2)
*36–45*	NA	34.0 (33.4–34.6)	98.6 (97.5–99.7)	6.0 (5.8–6.3)	1.1 (1.0–1.2)	139.8 (138.5–141.1)
*46–60*	NA	103.0 (102.0–104.1)	256.1 (254.4–257.7)	18.1 (17.6–18.5)	2.8 (2.6–2.9)	379.9 (377.9–381.9)
*61–80*	NA	442.0 (439.4–444.5)	666.3 (663.1–669.4)	99.8 (98.6–101.0)	15.2 (14.7–15.6)	1,223.2 (1219.0–1227.4)
*≥81*	NA	1,587.8 (1575.5–1600.1)	1,763.9 (1751.0–1776.9)	678.9 (670.8–686.9)	97.8 (94.7–100.8)	4,128.4 (4108.8–4148.0)

CI – confidence interval, NA – not applicable, RSV – respiratory syncytial virus

*Presented as n (%) unless specified otherwise. All age groups are presented in years.

†RSV mortality rates were not calculated because of limitations in testing frequency and diagnostic coverage, particularly among adults. These limitations compromise the comparability and reliability of RSV mortality estimates.

**Figure 3:**
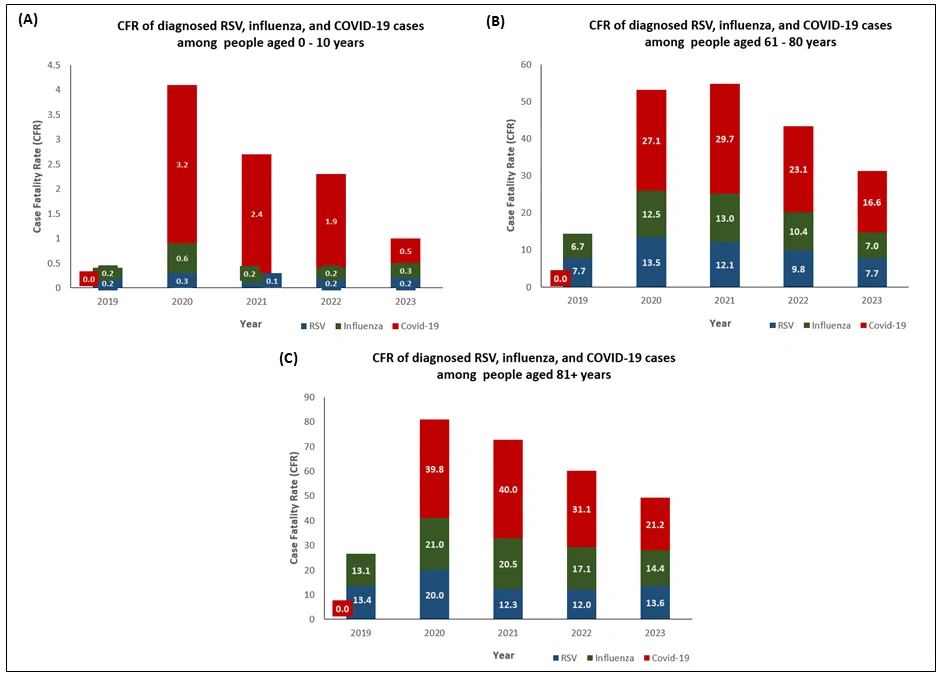
Case fatality rates for influenza and COVID-19 across age groups in Brazil, 2019–2023. **Panel A.** Case fatality rates among children aged 0–10 years. **Panel B.** Case fatality rates among adults aged 61–80 years. **Panel C.** Case fatality rates among adults aged 81 years or older.

Hospitalisation rates for influenza and COVID-19 were highest among individuals aged 81 years and older, at 151.3 and 7,803.9 per 100,000 population, respectively (Table S1 in the **Online Supplementary Document**). The CFR also increased with age for both viruses, reaching 14.4% (95% CI = 13.1–15.7) for influenza and 21.2% (95% CI = 19.9–22.4) for COVID-19 among individuals aged 81 years and older (**Figure 3**; Table S3 in the **Online Supplementary Document**). The impact of each virus on hospitalisations is described in Figure S1 in the **Online Supplementary Document**, and all percentages of hospitalisations by age group and year are available in Table S4 in the **Online Supplementary Document**.

Hospitalisation seasonality differed across high-risk age groups. Among children younger than two years, RSV demonstrated marked and recurrent seasonal peaks (Figure S3, Panel A in the **Online Supplementary Document**), whereas among adults aged 60 years or older and 70 years or older (Figure S3, Panels B and C in the **Online Supplementary Document**), seasonal patterns were driven primarily by influenza and COVID-19, with RSV contributing a smaller proportion of hospitalisations.

### Seasonality and regional variation

We observed distinct seasonal patterns for each virus (Figure S2, Panels A–C and Figure S3, Panel A–C in the **Online Supplementary Document**). RSV hospitalisations peaked between March and June, with the highest intensity in April. Influenza hospitalisations showed annual peaks in January across all regions. COVID-19 hospitalisations did not follow a clear seasonal pattern but instead showed multiple peaks corresponding to pandemic waves.

Regional analyses revealed that the Southeast and South regions of Brazil had the highest frequencies of hospitalisations and deaths for all three viruses (Figure S4 in the **Online Supplementary Document**). RSV hospitalisations peaked earlier in the North region, with a January increase, while the Northeast region showed pronounced influenza peaks during the same month. These regional differences in seasonal timing and burden were consistent throughout the study period.

The heatmap analysis further demonstrated distinct temporal and regional patterns in hospitalisations across pathogens. RSV showed consistent seasonal increases between March and June, with earlier activity in the North and Northeast regions, whereas influenza displayed more concentrated seasonal peaks, particularly in January. COVID-19 hospitalisations were characterised by marked temporal variability associated with successive pandemic waves and showed less consistent seasonality across regions (Figures S2–S4 in the **Online Supplementary Document**). Overall hospitalisation frequency was largely driven by COVID-19 during 2020-2022, with substantially lower levels observed thereafter (Figure S5 in the **Online Supplementary Document**).

## DISCUSSION

Here we analysed the epidemiological and seasonal patterns of laboratory-confirmed SARI cases attributed to RSV, influenza, and COVID-19 reported to the SIVEP-Gripe surveillance system in Brazil between 2019 and 2023. We observed clear differences among the three viruses in age distribution, mortality, hospitalisation rates, and seasonality, with important implications for public health planning. However, as our analysis was restricted to severe acute respiratory infection cases reported to SIVEP-Gripe and does not reflect patterns of infection at the community level, the results should not be interpreted as estimates of national incidence, prevalence, or population-level disease burden.

RSV was predominantly diagnosed in children under ten years of age, who accounted for more than 90% of reported cases annually. However, this concentration may reflect testing practices, rather than the true age distribution of the RSV disease. The limited availability and use of RSV testing in adults, together with lower sensitivity of diagnostic tests in older populations, suggest substantial underreporting [18]. This diagnostic gap is further supported by the growing proportion of RSV-related deaths among children, which increased from 19.2% in 2020 to 62.0% in 2023. Furthermore, children aged 0–10 years accounted for the large majority of reported RSV cases throughout the study period, ranging from 76.2% to 95.7% across years.

Interpretation of temporal trends between 2019 and 2023 must account for the major disruptions caused by the COVID‑19 pandemic, when changes in healthcare-seeking behaviour, testing availability, notification practices, and viral interference may have influenced the observed patterns. The reduced circulation of RSV and influenza during 2020–2021 likely reflected the effects of non-pharmaceutical interventions, school closures, and the prioritisation of SARS-CoV-2 testing rather than the true absence of these viruses [19,20]. Their subsequent resurgence may have been partly associated with increased population susceptibility following prolonged reductions in viral exposure, a phenomenon sometimes described as *‘*immune debt’.

Distinct age-specific patterns were observed across pathogens. Influenza cases and deaths were concentrated among individuals aged 61–80 years, while COVID-19 mortality was highest in individuals aged 81 years or older. In contrast, RSV hospitalisations occurred predominantly among young children, particularly those under two years of age. However, less frequent testing among adults than among infants may have contributed to this observed age distribution.

Influenza hospitalisations peaked in January, *i.e.* during the summer season in Brazil, consistent with circulation patterns in tropical and subtropical regions. RSV hospitalisations peaked between March and June, while COVID-19 showed no consistent seasonality, with peaks occurring at various times of the year. These differences may reflect Brazil’s diverse climatic zones, where viral circulation does not necessarily follow temperate-climate patterns.

The COVID-19 pandemic significantly influenced the epidemiology of other respiratory viruses. Studies suggest that SARS-CoV-2 disrupted influenza and RSV patterns, initially reducing cases before a subsequent resurgence [19]. This shift is partly due to ‘immune debt’, as reduced viral exposure during quarantine and distancing may have increased susceptibility once restrictions eased [20]. For RSV and influenza, the near absence of notifications in 2020–2021 likely reflected pandemic measures, including masking, school closures, mobility restrictions, and the overwhelming prioritisation of SARS‑CoV‑2 testing, rather than biological disappearance. The later resurgence of both viruses was consistent with the well‑documented post-pandemic rebound associated with reduced immunity and with the restoration of social contact patterns.

Our data revealed a variation in the burden of respiratory disease caused by RSV, influenza, and COVID-19 across age groups from 2019 to 2023. RSV cases increased more than fourfold from 2019 to 2023, with most cases occurring among children, while the burden among adults was likely underestimated due to limited testing [18]. Influenza cases increased notably in 2021 and 2022 and were concentrated among children and older adults aged 61–80 years. COVID-19 cases were most frequent in 2020 and 2021, primarily among older adults aged 61–80 years. These patterns highlight the vulnerability of children and older adults.

Geographic variation was evident in our sample, with the Southeast and South regions reporting the highest number of cases for all three viruses. RSV and influenza were most frequently reported in São Paulo and Paraná, while COVID-19 cases were most frequently reported in São Paulo and Minas Gerais. These differences may reflect disparities in population density, healthcare infrastructure, and surveillance performance, rather than differences in viral transmission alone, highlighting persistent regional inequalities in diagnostic access and reporting.

High mortality among children younger than two years of age and older adults may reflect their vulnerability to severe respiratory infections (Figure S3 in the **Online Supplementary Document**). Hospitalisation patterns showed a disproportionate burden among young children for RSV and influenza and among older adults for influenza and COVID-19, reinforcing the need for age-tailored prevention strategies. While limitations in RSV testing precluded reliable estimation of RSV hospitalisation rates outside paediatric populations, the observed age gradients across pathogens suggest distinct profiles of risk and disease severity.

Vaccination remains a cornerstone of prevention in these high-risk groups. Annual influenza vaccination and COVID-19 vaccination are essential for reducing severe outcomes among older adults [21]. The Brazilian Immunisation Society recommends RSV vaccination for elderly and other high-risk groups, while the National Immunization Programme has announced the inclusion of maternal RSV vaccination, which protects infants, as the only RSV strategy in the public programme by the end of 2025 [22,23]. Although we did not evaluate vaccination uptake or effectiveness, or vaccination programmes and their coverage, these findings could provide insights for future policy decisions aimed at reducing severe RSV outcomes.

Seasonality analysis revealed substantial heterogeneity in hospitalisation patterns across age groups and geographic regions of Brazil. The magnitude and duration of seasonal increases varied by pathogen, with RSV showing the sharpest rises, including up to a threefold increase in hospitalisations over a few months [24,25]. Regional differences were also evident, with influenza-related hospitalisations showing the greatest seasonal amplitude in the Northeast and COVID-19 burden being more variable in the North. Age-specific analyses demonstrated distinct epidemiological profiles, with RSV predominantly affecting young children, whereas seasonal increases among older adults extended over several months. These findings highlight important demographic and geographic differences in respiratory virus burden that may inform targeted prevention and healthcare planning. Moreover, seasonal timing should be interpreted in the context of Brazil’s diverse climatic zones, where viral circulation does not necessarily follow patterns typical of temperate climates [2,26].

The application of MSTL in our analysis allowed for the decomposition of overlapping and shifting seasonal patterns across pathogens in the context of pandemic-related disruptions, facilitating clearer isolation of seasonal signals than would be possible with single-season methods. However, the MSTL does not correct for surveillance artefacts or variations in testing intensity, and the results should therefore be interpreted as descriptive patterns.

### Study limitations

Several limitations must be acknowledged. Testing intensity varied substantially across pathogens, regions, and age groups. SARS-CoV-2 benefited from unprecedented expansion in testing, while RSV testing was largely limited to paediatric populations. As a result, we could not reliably estimate RSV hospitalisation and mortality rates, nor could we calculate the related CFR due to a highly selective denominator. Furthermore, CFR estimates for all pathogens are subject to surveillance bias, as they were calculated only among laboratory‑confirmed SARI cases and should not be interpreted as comparative measures of pathogen virulence. Additionally, deaths recorded in SIVEP-Gripe do not necessarily imply causal attribution to a specific pathogen, particularly among older adults with comorbidities or coinfections.

The study period was further characterised by major structural shifts in testing availability and health care utilisation. We did not undertake multivariable modelling because standardised testing denominators and longitudinal follow-up were unavailable in the SIVEP‑Gripe dataset. Given the supply-driven nature of diagnostic testing during the pandemic, regression approaches could have conflated testing intensity with epidemiological risk. Accordingly, we restricted our analysis to descriptive and time-series methods appropriate for surveillance data. Despite these limitations, the more consistent testing and reporting of influenza and COVID-19 allowed more reliable interpretation of their observed patterns. Time-series models may be better suited to estimate RSV burden among adults; for instance, Global Burden of Disease analyses suggest RSV-related hospitalisations among Brazilian adults are substantially underreported, particularly in tropical and subtropical regions [27].

## CONCLUSIONS

This national analysis of laboratory-confirmed SARI notifications in SIVEP-Gripe from 2019 to 2023 describes distinct age-specific and seasonal patterns for RSV, influenza, and COVID-19. However, the observed differences across pathogens should be interpreted cautiously, as testing intensity and diagnostic strategies varied substantially over time, across regions, and among age groups. Despite these limitations, the timing of seasonal peaks, particularly for RSV and influenza, may inform preparedness planning and future evaluations of preventive strategies. Strengthening and standardising diagnostic testing for respiratory viruses, especially RSV in adults, would improve the interpretation of surveillance data and support more robust epidemiological assessments.

## Additional material


Online Supplementary Document

## Data Availability

**Data availability:** The data used in this study are publicly available from the SIVEP-Gripe, maintained by the Brazilian Ministry of Health. The anonymised dataset, which contains records of severe acute respiratory infection cases reported across Brazil, is freely accessible through the Brazilian government’s OpenDataSUS platform: https://opendatasus.saude.gov.br/. No individual-level identifiers were used and all analyses complied with relevant ethical standards for research using secondary public data.

## References

[1] Pan American Health Organization. Influenza, SARS-CoV-2, RSV and other respiratory viruses. Available: https://www.paho.org/en/topics/influenza-sars-cov-2-rsv-and-other-respiratory-viruses. Accessed: 7 August 2026.

[2] HanageWPSchaffnerWBurden of acute respiratory infections caused by influenza virus, respiratory syncytial virus, and SARS-CoV-2 with consideration of older adults: a narrative review. Infect Dis Ther. 2025;14 Suppl 1:5–37. 10.1007/s40121-024-01080-439739200 PMC11724833

[3] PatelKPVunnamSRPatelPAKrillKLKorbitzPMGallagherJPTransmission of SARS-CoV-2: an update of current literature. Eur J Clin Microbiol Infect Dis. 2020;39:2005–11. 10.1007/s10096-020-03961-132638221 PMC7339796

[4] LuoWLiuQZhouYRanYLiuZHouWSpatiotemporal variations of “triple-demic” outbreaks of respiratory infections in the United States in the post-COVID-19 era. BMC Public Health. 2023;23:2452. 10.1186/s12889-023-17406-938062417 PMC10704638

[5] SwetsMCRussellCDHarrisonEMDochertyABLoneNGirvanMSARS-CoV-2 co-infection with influenza viruses, respiratory syncytial virus, or adenoviruses. Lancet. 2022;399:1463–4. 10.1016/S0140-6736(22)00383-X35344735 PMC8956294

[6] SantosCVValiatiNCMNoronhaTGPortoVBGPachecoAGFreitasLPThe effectiveness of COVID-19 vaccines against severe cases and deaths in Brazil from 2021 to 2022: a registry-based study. Lancet Reg Health Am. 2023;20:100465. 10.1016/j.lana.2023.10046536936517 PMC10010656

[7] Sociedade Brasileira de Imunizações. Vacinas VSR (vírus sincicial respiratório). 23 January 2026. Available: https://familia.sbim.org.br/vacinas/vacinas-disponiveis/vacinas-vsr-virus-sincicial-respiratorio. Accessed: 7 August 2026.

[8] FreitasLPCodeçoCTBastosLSVillelaDAMCruzOGPachecoAGEvaluation of the design of the influenza-like illness sentinel surveillance system in Brazil. Cad Saude Publica. 2024;40:e00028823. 10.1590/0102-311xen02882339082558 PMC11321611

[9] Ministério da Saúde. Guia de vigilância integrada da COVID-19, influenza e outros vírus respiratórios de importância em saúde pública. Brasilia, Brazil: Ministério da Saúde; 2024. Available: https://www.gov.br/saude/pt-br/centrais-de-conteudo/publicacoes/guias-e-manuais/2024/guia-vigilancia-integrada-da-covid-19-influenza-e-outros-virus-respiratorios-de-importancia-em-saude-publica. Accessed: 7 August 2026.

[10] Secretaria da Saúde da Bahia. Guia rápido SIVEP Gripe. Salvador, Brazil: Secretaria da Saúde da Bahia; 2021. Available: https://www.saude.ba.gov.br/wp-content/uploads/2021/05/GUIA-RAPIDO-SIVEP-GRIPE-atualizado-em-maio_2021.pdf. Accessed: 7 August 2026.

[11] Brazil Ministry of Health. SRAG 2019 a 2026 – banco de dados de síndrome respiratória aguda grave. 2026. Available: https://dadosabertos.saude.gov.br/dataset/srag-2019-a-2026. Accessed: 7 August 2026.

[12] Instituto Brasileiro de Geografia e Estatística. Malhas territoriais 2023. 2023. Available: https://www.ibge.gov.br/geociencias/organizacao-do-territorio/malhas-territoriais/15774-malhas.html. Accessed: 7 August 2026.

[13] Bandara K Hyndman RJ Bergmeir C MSTL: a seasonal-trend decomposition algorithm for time series with multiple seasonal patterns. Int J Oper Res. 2025;52:79–98. 10.1504/IJOR.2025.143957

[14] Trull O García-Díaz JC Peiró-Signes A Multiple seasonal STL decomposition with discrete-interval moving seasonalities. Appl Math Comput. 2022;433:127398. 10.1016/j.amc.2022.127398

[15] Sohrabbeig A Ardakanian O Musilek P Decompose and conquer: time series forecasting with multiseasonal trend decomposition using Loess. Forecasting. 2023;5:684–96. 10.3390/forecast5040037

[16] WilliamsJRYangRCliffordJLWatsonDCampbellRGetnetDFunctional heatmap: an automated and interactive pattern recognition tool to integrate time with multi-omics assays. BMC Bioinformatics. 2019;20:81. 10.1186/s12859-019-2657-030770734 PMC6377781

[17] WiemkenTLKhanFPuzniakLYangWSimmeringJPolgreenPSeasonal trends in COVID-19 cases, hospitalizations, and mortality in the United States and Europe. Sci Rep. 2023;13:3886. 10.1038/s41598-023-31057-136890264 PMC9994397

[18] BegierEAliabadiNRamirezJAMcGeerALiuQCarricoRDetection by nasopharyngeal swabs alone underestimates respiratory syncytial virus-related hospitalization incidence in adults: the Multispecimen Study’s final analysis. J Infect Dis. 2025;232:e126–e136. 10.1093/infdis/jiaf20440250971 PMC12308649

[19] Magalhães BronzeKdos SantosURBarbosa CostaGSeváADGuimarães KersulMSacramento PintoCThe impact of the COVID-19 pandemic on the clinical and epidemiological profile of severe acute respiratory infection in Bahia, Brazil: a comparative analysis of pre- and post-pandemic trends. Viruses. 2025;17:389. 10.3390/v1703038940143317 PMC11946068

[20] Azevedo JVV Santos CAC Silva MT Olinda RA Santos DAS Análise das variações climáticas na ocorrência de doenças respiratórias por influenza em idosos na região metropolitana de João Pessoa - PB. Soc Nat. 2017;29:123–35. 10.14393/SN-v29n1-2017-8

[21] SinghalSKumarPSinghSSahaSDeyABClinical features and outcomes of COVID-19 in older adults: a systematic review and meta-analysis. BMC Geriatr. 2021;21:321. 10.1186/s12877-021-02261-334011269 PMC8133052

[22] Pérez Marc G Vizzotti C Fell DB Di Nunzio L Olszevicki S Mankiewicz SW Real-world effectiveness of RSVpreF vaccination during pregnancy against RSV-associated lower respiratory tract disease leading to hospitalisation in infants during the 2024 RSV season in Argentina (BERNI study): a multicentre, retrospective, test-negative, case-control study. Lancet Infect Dis. 2025;25:1044–54. 10.1016/S1473-3099(25)00156-2 40339585

[23] Symes R Whitaker HJ Ahmad S Arnold D Banerjee S Evans CM Vaccine effectiveness of a bivalent respiratory syncytial virus pre-F vaccine against RSV-associated hospitalisation among adults aged 75-79 years in England: a multicentre, test-negative, case–control study. Lancet Infect Dis. 2026;26:229–38. 10.1016/S1473-3099(25)00546-8 41167207

[24] ViannaLASiqueiraMMVolpiniLPBLouroIDResendePCSeasonality, molecular epidemiology, and virulence of respiratory syncytial virus (RSV): a perspective into the Brazilian Influenza Surveillance Program. PLoS One. 2021;16:e0251361. 10.1371/journal.pone.025136134003843 PMC8130917

[25] Centers for Disease Control and Prevention. Respiratory syncytial virus infection (RSV). 2025. Available: https://www.cdc.gov/rsv/hcp/clinical-overview/index.html. Accessed: 7 August 2026.

[26] ShiTMcAllisterDAO’BrienKLSimoesEAFMadhiSAGessnerBDGlobal, regional, and national disease burden estimates of acute lower respiratory infections due to respiratory syncytial virus in young children in 2015: a systematic review and modelling study. Lancet. 2017;390:946–58. 10.1016/S0140-6736(17)30938-828689664 PMC5592248

[27] BurkartKLiangCRaffertyQGillespieCWMcLaughlinSOrosARespiratory syncytial virus-attributable hospitalizations among adults in high- and middle-income countries: application of the Global Burden of Disease framework. EClinicalMedicine. 2025;85:103292. 10.1016/j.eclinm.2025.10329240599872 PMC12209947

