# Supplementary material for: Seasonal dynamics and burden of respiratory syncytial virus, influenza, and COVID-19 in Brazil: a national surveillance study (2019–2023)
Source: J Glob Health. 2026 Aug 28;16:04251. doi: 10.7189/jogh.16.04251 (PMC13521488; doi:10.7189/jogh.16.04251)
Supplement: Supplementary file 1 [file jogh-16-04251-s001.pdf]

Supplement to: Julian GS, Spinardi JR, Flora AP, Valluri SR, d'Hellencourt FL, Liang C, Begier E, Berra TZ, Cortez LV, Vitorasso R, Kyaw MH. Seasonal dynamics and burden of respiratory syncytial virus, influenza, and COVID-19 in Brazil: a national surveillance study (2019–2023). J Glob Health. 2026;16:04251.

Supplementary Tables

Table S1 - Hospitalization rate due to respiratory infections by Influenza and COVID-19 according to age group and year of notification, Brazil (2019-2023)

	2019	2020	2021	2022	2023	Overall
**INFLUENZA**						
**Hospitalization rate* (CI95%)**						
**Age Group**						
0 to 10 years	7.1 (6.8-7.4)	2.2 (2.0-2.4)	6.0 (5.7-6.3)	10.8 (10.5-11.2)	17.5 (17.0-18.0)	43.6 (42.7-44.4)
11 to 20 years	1.3 (1.2-1.4)	0.5 (0.4-0.6)	1.9 (1.7-2.1)	2.9 (2.6-3.0)	3.5 (3.3-3.7)	10.0 (9.66-10.4)
21 to 35 years	1.8 (1.6-1.9)	0.8 (0.7-0.9)	2.6 (2.4-2.7)	1.8 (1.7-1.9)	1.4 (1.3-1.5)	8.4 (8.1-8.6)
36 to 45 years	2.1 (1.9-2.3)	0.7 (0.7-0.8)	2.2 (2.1-2.4)	1.7 (1.6-1.8)	2.2 (2.0-2.3)	9.0 (8.6-9.3)
46 to 60 years	3.0 (2.8-3.1)	0.8 (0.7-0.9)	2.8 (2.7-3.0)	2.7 (2.5-2.8)	2.8 (2.6-2.9)	12.0 (11.7-12.4)
61 to 80 years	4.6 (4.4-4.9)	1.4 (1.2-1.5)	12.2 (11.8-12.7)	11.7 (11.3-12.1)	7.3 (7.0-7.6)	37.3 (36.6-38.1)
81+ years	11.9 (10.8-12.9)	4.0 (3.4-4.6)	59.7 (57.3-62.1)	50.7 (47.9-52.3)	25.7 (24.1-27.2)	151.3 (147.4-155.0)
**COVID-19**						
**Hospitalization rate* (CI95%)**						
**Age Group**						
0 to 10 years	-	35.1 (34.4-35.7)	48.6 (47.8-49.4)	62.2 (61.3263.1)	27.8 (27.2-28.4)	173.7 (172.2-175.2)
11 to 20 years	-	20.5 (20.0-21.1)	30.4 (29.7-31.0)	15.3 (14.8-15.7)	3.8 (3.6-4.1)	70.1 (69.1-71.0)
21 to 35 years	-	105.2 (104.3-106.2)	228.8 (227.4-230.2)	30.7 (30.2-31.2)	6.0 (5.7-6.2)	370.8 (369.0-372.5)
36 to 45 years	-	257.6 (255.8-259.3)	587.8 (585.1-590.4)	42.4 (41.7-43.1)	8.1 (7.8-8.4)	895.9 (892.7-899.2)
46 to 60 years	-	474.7 (472.5-476.9)	996.4 (993.2-999.6)	83.3 (82.4-84.2)	15.7 (15.3-16.1)	1570.1 (1566.0-1574.1)
61 to 80 years	-	1057.6 (1053.7-1061.5)	1563.0 (1558.3-1567.8)	330.9 (328.6-333.1)	66.5 (65.5-67.5)	3018.0 (3011.4-3024.6)
81+ years	-	2598.5 (2582.9-2614.2)	3072.1 (3055.1-3089.1)	1766.3 (1753.3-1779.2)	366.9 (361.0-372.9)	7803.9 (7777.5-7830.2)

** Hospitalization rate per 100.000 inhab.*

Note: RSV hospitalization rates were not calculated due to limitations in testing frequency and diagnostic coverage, especially among adults. These limitations compromise the comparability and reliability of RSV hospitalization estimates.

Table S2. Percentage of deaths per age group among all deaths due to RSV, Influenza and COVID among people of the respective age group, Brazil (2019-2023)

	2019	2020	2021	2022	2023	Overall
**RSV**						
**Age Group**						
0 to 10 years	54.6 %	3.3 %	14.8 %	19.2 %	47.5 %	22.5 %
11 to 20 years	6.0 %	0.0 %	1.0 %	1.0 %	5.1 %	1.0 %
21 to 35 years	17.0 %	0.1 %	0.0 %	0.5 %	2.5 %	0.2 %
36 to 45 years	6.7 %	0.0 %	0.0 %	0.6 %	1.3 %	0.1 %
46 to 60 years	7.1 %	0.0 %	0.0 %	0.5 %	1.4 %	0.0 %
61 to 80 years	11.9 %	0.0 %	0.0 %	0.4 %	1.8 %	0.1 %
81+ years	19.5 %	0.0 %	0.1 %	0.3 %	1.5 %	0.1 %
**INFLUENZA**						
**Age Group**						
0 to 10 years	46.3 %	2.9 %	4.0 %	5.9 %	22.8 %	11.0 %
11 to 20 years	93.9 %	1.8 %	3.2 %	7.9 %	47.4 %	8.0 %
21 to 35 years	82.9 %	0.5 %	0.5 %	4.1 %	19.0 %	1.3 %
36 to 45 years	93.2 %	0.2 %	0.2 %	2.7 %	18.3 %	0.8 %
46 to 60 years	92.8 %	0.1 %	0.1 %	2.6 %	13.9 %	0.6 %
61 to 80 years	88.0 %	0.0 %	0.3 %	2.3 %	8.4 %	0.6 %
81+ years	80.5 %	0.1 %	0.9 %	2.1 %	6.3 %	1.0 %
**COVID-19**						
**Age Group**						
0 to 10 years	-	93.7 %	81.1 %	74.8 %	29.6 %	66.3 %
11 to 20 years	-	98.1 %	95.7 %	91.0 %	47.4 %	90.9 %
21 to 35 years	-	99.3 %	99.4 %	95.3 %	78.3 %	98.3 %
36 to 45 years	-	99.7 %	99.7 %	96.5 %	80.3 %	99.0 %
46 to 60 years	-	99.8 %	99.7 %	96.8 %	84.6 %	99.2 %
61 to 80 years	-	99.8 %	99.5 %	97.2 %	89.6 %	99.2 %
81+ years	-	99.8 %	98.9 %	97.5 %	92.1 %	98.8 %

Table S3. Case Fatality Rate (CFR) due to Influenza and SARS-Cov-2 for each year of the study period, according to age group

	2019		2020		2021		2022		2023	
	**rate**	**CI 95%**	**rate**	**CI 95%**	**rate**	**CI 95%**	**rate**	**CI 95%**	**rate**	**CI 95%**
**Influenza**										
**Age group - n (%)**										
0 to 10 years	0.2	(0.1 - 0.4)	0.6	(0.3 - 0.9)	0.2	(0.1 - 0.4)	0.2	(0.1 - 0.3)	0.3	(0.2 - 0.5)
11 to 20 years	0.5	(0.1 - 0.8)	0.9	(0.3 - 1.5)	1.2	(0.4 - 1.9)	0.5	(0.2 - 0.8)	0.2	(0.0 - 0.4)
21 to 35 years	0.9	(0.5 - 1.4)	1.6	(1.1 - 2.1)	1.6	(1.0 - 2.3)	0.9	(0.5 - 1.2)	0.8	(0.4 - 1.2)
36 to 45 years	2.6	(1.7 - 3.5)	3.9	(3.2 - 4.7)	4.0	(3.0 - 5.1)	2.1	(1.5 - 2.8)	1.7	(1.0 - 2.4)
46 to 60 years	4.3	(3.4 - 5.2)	6.2	(5.5 - 6.9)	7.2	(6.1 - 8.2)	5.1	(4.3 - 5.9)	3.6	(2.7 - 4.4)
61 to 80 years	6.7	(5.9 - 7.6)	12.5	(11.7 - 13.4)	13.0	(12.0 - 14.1)	10.4	(9.6 - 11.1)	7.0	(6.2 - 7.8)
81+ years	13.1	(11.7 - 14.6)	21.0	(19.4 - 22.6)	20.5	(18.7 - 22.4)	17.1	(16.0 - 18.2)	14.4	(13.1 - 15.7)
**Sars-Cov-2**										
**Age group - n (%)**										
0 to 10 years	-		3.2	(2.8 - 3.5)	2.4	(2.1 - 2.6)	1.9	(1.6 - 2.1)	0.5	(0.2 - 0.8)
11 to 20 years	-		5.3	(4.7 - 5.9)	5.7	(5.2 - 6.2)	4	(3.3 - 4.7)	1.2	(0.0 - 2.4)
21 to 35 years	-		6.9	(6.6 - 7.2)	8.8	(8.6 - 9.0)	6.5	(6.0 - 7.0)	6.3	(4.7 - 8.0)
36 to 45 years	-		9.4	(9.1 - 9.6)	12.4	(12.2 - 12.6)	10.7	(10.1 - 11.3)	9.7	(7.6 - 11.7)
46 to 60 years	-		14.7	(14.5 - 14.9)	18.3	(18.2 - 18.5)	15.9	(15.4 - 16.4)	12.9	(11.4 - 14.4)
61 to 80 years	-		27.1	(26.9 - 27.3)	29.7	(29.5 - 29.8)	23.1	(22.8 - 23.5)	16.6	(15.6 - 17.5)
81+ years	-		39.8	(39.4 - 40.2)	40.0	(39.7 - 40.33)	31.1	(30.6 - 31.6)	21.2	(19.9 - 22.4)

Table S4. Frequency of hospitalizations per age group and year, Brazil (2019-2023)

	2019	2020	2021	2022	2023	Overall
**RSV**						
**Age Group**						
0 to 10 years	5512 (98.2%)	1289 (98.0%)	12819 (98.2%)	17048 (97.6%)	25968 (96.0%)	62636 (97.1%)
11 to 20 years	64 (98.5%)	16 (94.1%)	133 (94.3%)	106 (96.4%)	174 (92.5%)	493 (94.6%)
21 to 35 years	98 (98.0%)	47 (78.3%)	102 (94.4%)	99 (90.0%)	77 (93.9%)	423 (91.9%)
36 to 45 years	56 (100%)	48 (87.3%)	90 (93.7%)	75 (93.7%)	70 (92.1%)	339 (93.4%)
46 to 60 years	113 (96.6%)	76 (97.4%)	195 (92.8%)	189 (92.6%)	160 (98.7%)	733 (95.1%)
61 to 80 years	160 (98.1%)	136 (95.1%)	409 (97.1%)	562 (95.2%)	412 (96.7%)	1679 (96.3%)
81+ years	112 (99.1%)	50 (87.7%)	228 (95.8%)	352 (94.4%)	257 (94.5%)	999 (94.9%)
**INFLUENZA**						
**Age Group**						
0 to 10 years	2049 (98.1%)	643 (96.2%)	1718 (94.6%)	3127 (97.0%)	5049 (91.6%)	12586 (94.6%)
11 to 20 years	366 (95.1%)	141 (91.5%)	526 (89.4%)	789 (92.9%)	980 (77.3%)	2802 (86.4%)
21 to 35 years	822 (95.7%)	367 (88.2%)	1170 (82.6%)	828 (86.9%)	649 (68.9%)	3836 (83.6%)
36 to 45 years	668 (96.8%)	233 (86.0%)	695 (83.2%)	536 (87.1%)	684 (76.6%)	2816 (85.2%)
46 to 60 years	1089 (97.0%)	283 (90.4%)	1032 (87.9%)	983 (91.7%)	1019 (86.8%)	4406 (90.7%)
61 to 80 years	1195 (97.6%)	357 (93.4%)	3113 (91.3%)	2984 (93.6%)	1879 (94.2%)	9528 (93.4%)
81+ years	469 (98.1%)	155 (95.7%)	2339 (91.1%)	1961 (92.8%)	1005 (93.9%)	5929 (92.8%)
**COVID-19**						
**Age Group**						
0 to 10 years	-	10047 (91.2%)	13949 (94.3%)	17907 (96.1%)	8015 (96.3%)	49918 (94.6%)
11 to 20 years	-	5736 (83.6%)	8447 (91.5%)	4233 (92.8%)	1074 (94.4%)	19490 (89.4%)
21 to 35 years	-	48367 (85.3%)	105511 (94.9%)	14043 (91.3%)	2733 (91.2%)	170654 (91.6%)
36 to 45 years	-	80898 (91.2%)	185101 (95.8%)	13289 (92.4%)	2557 (92.2%)	281845 (94.3%)
46 to 60 years	-	173367 (93.8%)	364349 (95.8%)	30364 (93.9%)	5740 (93.7%)	573820 (95.1%)
61 to 80 years	-	269329 (94.3%)	398456 (95.2%)	84405 (94.9%)	16993 (95.0%)	769183 (94.8%)
81+ years	-	101324 (92.6%)	119918 (93.8%)	69312 (94.9%)	14419 (95.3%)	304973 (93.7%)

Table S5. Percentage of hospitalizations per age group among all hospitalizations due to RSV, Influenza and COVID among people of the respective age group, Brazil (2019-2023)

	2019	2020	2021	2022	2023	Overall
**RSV**						
**Age Group**						
0 to 10 years	72.9 %	10.7 %	45.0 %	44.7 %	66.5 %	50.0%
11 to 20 years	15.8 %	0.2 %	1.4 %	2.0 %	7.8 %	2.1 %
21 to 35 years	10.6 %	0.1 %	0.1 %	0.6 %	2.2 %	0.2 %
36 to 45 years	7.7 %	0.0 %	0.0 %	0.5 %	2.1 %	0.1 %
46 to 60 years	9.4 %	0.0 %	0.0 %	0.6 %	2.3 %	0.1 %
61 to 80 years	11.8 %	0.0 %	0.1 %	0.6 %	2.1 %	0.2 %
81+ years	19.2 %	0.0 %	0.1 %	0.4 %	1.6 %	0.3 %
**INFLUENZA**						
**Age Group**						
0 to 10 years	27.1 %	5.3 %	6.0 %	8.21 %	12.9 %	10.0 %
11 to 20 years	85.1 %	2.3 %	5.7 %	15.3 %	43.9 %	12.3 %
21 to 35 years	89.3 %	0.7 %	1.1 %	5.5 %	18.7 %	2.1 %
36 to 45 years	92.2 %	0.2 %	0.3 %	3.8 %	20.6 %	0.9 %
46 to 60 years	90.6 %	0.1 %	0.2 %	3.1 %	14.7 %	0.7 %
61 to 80 years	88.1 %	0.1 %	0.7 %	3.3 %	9.7 %	1.2 %
81+ years	80.7 %	0.1 %	1.9 %	2.7 %	6.4 %	1.9 %
**COVID-19**						
**Age Group**						
0 to 10 years	-	83.8 %	48.9 %	47.0 %	20.5 %	39.8 %
11 to 20 years	-	97.3 %	92.7 %	82.5 %	48.2 %	85.5 %
21 to 35 years	-	99.1 %	98.8 %	93.8 %	79.0 %	97.5 %
36 to 45 years	-	99.6 %	99.5 %	95.6 %	77.2 %	98.8 %
46 to 60 years	-	99.7 %	99.6 %	96.2 %	82.9 %	99.1 %
61 to 80 years	-	99.8 %	99.1 %	96.9 %	88.1 %	98.5 %
81+ years	-	99.8 %	97.9 %	96.7 %	91.9 %	97.7 %

Supplementary Figures

Figure S1 - Percentage of hospitalizations reported by pathogen per age group among all hospitalizations due to RSV, influenza and COVID among people of the respective age group, Brazil (2019-2023)
Legend: *Graphs showing the percentage of hospitalizations attributed to respiratory syncytial virus (RSV), influenza and COVID-19 between 2019 and 2023. A) General distribution of hospitalizations including all ages; B) Distribution of hospitalizations among children aged 0 to 10; C) Distribution of hospitalizations among adults aged 61 to 80; D) Distribution of hospitalizations among elderly people aged 81 or over.*

Figure S2 - Frequency and seasonality of hospitalizations due to RSV, influenza and COVID-19, Brazil (2019-2023)
Caption: (A) Time series and seasonality of the frequency of hospitalizations due to RSV, Influenza and COVID-19. (B) Heatmap of seasonality of hospitalizations due to RSV, influenza and COVID-19.
Color gradient in the heatmap represents the intensity of the seasonal component, by convention, warm colors indicate higher seasonal contribution, while cool colors indicate lower contribution.

Figure S3 - Frequency and seasonality of hospitalizations due to RSV, influenza and COVID-19, according to age group, Brazil (2019-2023)
Caption: (A) Frequency and seasonality of hospitalizations due to RSV, Influenza and COVID-19, in infants under 2 years old. (B) Frequency and seasonality of hospitalizations due to RSV, Influenza and COVID-19, in people over 60 years old. (C) Frequency and seasonality of hospitalizations due to RSV, Influenza and COVID-19, in people over 70 years old.

Figure S4 - Frequency and seasonality of hospitalizations due to RSV, influenza and COVID-19, according to region, Brazil (2019-2023)
Caption: Color gradient in the heatmap represents the intensity of the seasonal component, by convention, warm colors indicate higher seasonal contribution, while cool colors indicate lower contribution.
Note: Lower RSV testing frequency in adults as compared to infants and for COVID19 and influenza limit the comparability of results for RSV disease.

Figure S5 - Frequency of hospitalizations due to RSV, Influenza and SARS-CoV-2 over the period 2019-2023
Caption: Time series of hospitalization frequency associated with RSV, influenza, and SARS-COV-2 from 2019 to 2023. The black line shows observed frequency, and the purple dashed line depicts the estimated seasonal component. Dashed vertical lines mark the beginning of each year.
